# Relationship between Tibia Lead and Cumulative Blood Lead Index: Schwartz et al. Respond

**DOI:** 10.1289/ehp.10778R

**Published:** 2008-03

**Authors:** Brian S. Schwartz, Howard Hu, Stephen J. Rothenberg, Andrew C. Todd

**Affiliations:** Johns Hopkins University, Baltimore, Maryland, E-mail: bschwart@jhsph.edu; University of Michigan, Ann Arbor, Michigan; Centro de Investigación y de Estudios, Avanzados del I.P.N. Unidad de Mérida, Mérida, Yucatán, México; Mount Sinai School of Medicine, New York, New York

We thank Healey et al. for their thoughtful comments on our papers in the mini-monograph on adult lead exposure (Hu et al. 2007; Schwartz and Hu 2007). We agree with the points they raised concerning uncertainties in the relation of cumulative blood lead index (CBLI) with tibia lead, the need to address the possibility that the slope of the relation may not be constant across the range of tibia lead values, and uncertainty about how sex may influence the relation, as most data were derived from studies of men. Given the changing metabolism of bone across the life span, age must ultimately be factored in as well.

Healey et al. posit that the relation of CBLI with tibia lead may be nonlinear by presenting summary data from eight studies; they show that the estimated slope of the CBLI and tibia lead relation is relatively low in studies with lower mean tibia lead levels, whereas the estimated slope appears to be higher in studies with higher mean tibia lead levels. A problem with this argument is that it is prone to the ecologic fallacy of using summary data of CBLI and mean tibia lead from groups across studies to make inferences in individuals about the relation of CBLI with tibia lead. All of the literature evaluating relations of CBLI with tibia lead is based on measurements in only approximately 500 subjects. A rigorous assessment for possible nonlinearities in such a relation would require a pooled analysis of the original data, not an ecologic analysis of the summary results across studies (Lanphear et al. 1998). Several statistical techniques could then be used to evaluate possible departures from linearity in the CBLI versus tibia lead relation using the individual level data.

Concerning the range of slopes we reported, we wrote “Each study also reported sample size and the SE of the slope, which, across the studies, ranged from 0.028 to 0.067” (Hu et al. 2007). Healey et al. correctly report that the slopes ranged from 0.022 to 0.10 μg/g per μg-years/dL across the eight studies they included. This discrepancy is easily explained. Concerning the high end of their range, we chose not to use the Armstrong et al. slope estimates for 1983 or 1988 (both 0.10, based on 15 and 11 subjects, respectively), instead relying on their estimate (0.052) for the 11 subjects for both 1983 and 1988 (Armstrong et al. 1992). Concerning the low end of their range (0.022), Gerhardsson et al. (1993) did not present an SE of the slope, which is needed, along with the slope estimate, for use in a meta-analysis.

Given the ecologic fallacy issue discussed above, we respectfully disagree with the recommendation of Healey et al. that we should rely only on the two studies that reported the smallest slopes for the relation of CBLI with tibia lead (Gerhardsson 1993; Erkkilä 1992) simply because, as they note, these studies had average tibia lead levels closest to the 15 μg/g tibia lead limit we proposed (Schwartz and Hu 2007). In addition, we believe that relying on only two relatively small studies does not provide sufficient margin of safety for lead-exposed workers to accept Healey et al.’s recommendation at this time. Because our primary goal is preventing departures from health associated with cumulative lead dose in adults exposed to lead, we instead choose to rely on an estimate derived from a weighted mean across studies. At the present time, we believe we are justified in standing by our recommendation of a CBLI of 200–400 μg-years/dL.
